# Coupling coordination of digital village construction and high-quality agricultural development in China

**DOI:** 10.1371/journal.pone.0319090

**Published:** 2025-02-07

**Authors:** Yuan Tian, Yuxi Zhou

**Affiliations:** School of Economics and Management, Shandong Agricultural University, Taian, China; Shandong University of Science and Technology, CHINA

## Abstract

This study aims to investigate the coupling coordination relationship between digital village construction and high-quality agricultural development in China, along with influencing factors, in order to provide policy recommendations for fostering the concurrent advancement of these two domains. Utilizing coupling coordination degree model, spatial econometric model and other methods, this paper analyzes the panel data of 31 provinces (municipalities and autonomous regions) in China spanning from 2011 to 2022. The key findings indicate the following: Firstly, there has been an overall enhancement in the levels of digital village construction and high-quality agricultural development across all Chinese provinces, with the eastern region exhibiting a higher developmental status compared to the central and western regions. Secondly, the coupling coordination degree has shown a collective improvement, albeit with variations in coordination levels among different regions; however, the regional disparities are gradually narrowing. Thirdly, the level of economic development and agricultural financial expenditure emerges as significant factors influencing the degree of coupling coordination, demonstrating not only direct positive effects but also positive spatial spillover effects. Finally, this study proposes recommendations to promote sustainable agricultural development, such as setting up agricultural technology innovation platforms, establishing digital agricultural production bases, and training farmers in digital skills in order to mitigate regional disparities and foster mutual advancement across all regions.

## 1 Introduction

High-quality development has emerged as a new imperative for China’s economic and social development. In this context, as an important industry, achieving high-quality development in agriculture is an important area in the process of advancing overall economic and societal high-quality development. The advancement of high-quality agricultural development holds profound significance for China’s comprehensive promotion of rural revitalization and the cultivation of a robust agricultural sector [1]. However, China’s agriculture continues to grapple with challenges such as inadequate resource utilization and limited digital literacy among farmers. In the face of these challenges, it is necessary to drive the transition of agricultural production methods and rely on digital technology to achieve high-quality agricultural development [2]. Digital village construction stands as a key strategy in the rural revitalization process, characterized by employing digital economy principles to enhance efficiency and quality improvements across agriculture and rural areas through technological innovation, thereby fostering high-quality agricultural development.

Governments worldwide have underscored the significance of digital technology in agriculture, recognizing its role in fostering high-quality agricultural development. For example, MacPherson et al. argue that digital agriculture has the potential to enhance the sustainability of the entire food system. Their examination of policy frameworks in Germany and Europe underscores the pivotal role of digital agriculture in advancing sustainable development objectives [3]. The case of Tanzania proves that advanced digital technologies for agriculture contribute significantly to sustainable agriculture, offering a pathway to alleviate extreme poverty and food insecurity among a majority of smallholder farmers globally [4]. Delving into the Brazilian agro-ecosystem, Engas et al. find that digital technology advancement affects agricultural development, with the technological framework of the participants plays an important role in the development of digital agriculture [5]. Johnson et al. argue that digital transformation of agriculture can improve food security. Drawing on the example of Jamaica, a small island developing state, they analyze the challenges faced by the digital transformation of agriculture and propose improvement measures [6]. The interconnection between digital village construction and high-quality agricultural development is empirically established through these studies.

From the perspective of synergy theory, there is an interaction between digital village construction and high-quality agricultural development. On the one hand, digital village construction emphasizes utilizing digital technologies to elevate informatization levels, foster smart agricultural practices and enhance farmers’ digital competencies. The application of digital technology can enhance the efficiency of agricultural production, optimize the allocation of rural resources and improve the rural living environment, etc., which plays an important role in achieving high-quality agricultural development [7, 8]. On the other hand, the essence of high-quality agricultural development lies in improving the quality and efficiency of agricultural products and accelerating the pace of agricultural modernization in order to achieve sustainable development of agriculture. The development of digital agricultural production, digital rural services, etc. is precisely to achieve this goal [9]. By providing digital, informatized and intelligent support for high-quality agricultural development, digital village construction promotes sustainable agricultural development and the realization of agricultural modernization objectives. Thus, the intricate connection between digital village construction and high-quality agricultural development underscores their mutual promotion and influence. Based on the above, analyzing the coupling coordination relationship between digital village construction and high-quality agricultural development not only fosters a scholarly comprehension of their interrelationship but also furnishes theoretical underpinnings for fortifying digital village construction initiatives and materializing high-quality agricultural development goals.

This study aims to explore the coupling coordination relationship between digital village construction and high-quality agricultural development. It focuses on the following issues. Firstly, it aims to establish an indicator evaluation system for both digital village construction and high-quality agricultural development to gauge their respective levels. Secondly, we explore the interactive relationship by calculating the degree of coupling coordination between them, and analyze the evolutionary characteristics of coupling coordination with the help of kernel density estimation. Finally, we use spatial econometric model to reveal the factors affecting the coupling and coordination between the two, and propose policy recommendations. The novelty of this study is mainly reflected in the fact that it considers the interaction between digital village construction and high-quality agricultural development, analyzes the coupling and coordination degree between the two, and reveals the influencing factors. Existing studies mainly focus on the measurement and evaluation of the level of digital village construction and high-quality agricultural development, as well as the impact of digitalization on agricultural development. These studies only analyze from a single perspective, exploring individual characteristics or causality without fully acknowledging the intricate interactive relationship. Therefore, this paper fills the gap in previous studies by analyzing the relationship between the two. In summary, our contributions can be summarized as follows: Firstly, this paper innovatively analyzes the coupling coordination relationship between China’s digital village construction and high-quality agricultural development. On the basis of evaluating the level of digital village construction and high-quality agricultural development in China, this study thoroughly explores the internal mechanism of the coupling coordination relationship between them and influencing factors, which enriches and supplements the research in the field of digital village construction and high-quality agricultural development. Secondly, it comprehensively analyzes the internal mechanism and evolutionary characteristics of the coordination relationship between the two, offering valuable insights for future studies on their interaction. Furthermore, this study provides both theoretical and practical guidance for advancing the digital village construction and high-quality agricultural development in China. Finally, the spatial econometric model is utilized to reveal the influencing factors of the coordination relationship and provide scientific basis for the formulation of relevant policies, and holds significant implications for the mutual promotion of the two and the formation of a virtuous circle.

The structure of this paper is as follows: Section 2 introduces an overview of research progress in digital village construction and high-quality agricultural development. Section 3 outlines the methodology and indicator system. Section 4 presents the results and analysis. Section 5 presents discussion. And conclusions and policy recommendations is provided in section 6.

## 2 Literature review

For the research on digital village construction and high-quality agricultural development, we can summarize the following three aspects by combing the relevant literature.

### 2.1 Research on digital village construction

Scholars mainly focus on research in the fields of the concept, level measurement and evaluation of digital village construction. For the research on the concept of digital village construction, scholars have explained the rich connotation of digital village construction from multiple perspectives. Firstly, in terms of the essence of digital village construction, it is the process of applying digital technology to village construction and governance, which can improve the level of public services and promote urban-rural integration [10]. Secondly, the application of digital technology can promote the upgrading of village industries, especially the development of digital agriculture. Scholars have sorted out the dynamic development process of digital agriculture and smart agriculture [11], pointing out that digital agriculture can increase productivity, reduce resource wastage, achieve sustainable development and further guarantee food security [6]. In addition, digital inclusive finance is also powering the building of digital villages scholars believe that digital inclusive finance can promote village economic growth, improve the way of income distribution among farmers, and promote the integrated development of village industries [12, 13]. For the study of measuring and evaluating the level of digital village construction, scholars have used a variety of methods to measure the level of digital village construction from multiple perspectives. For example, the indicator system is constructed from the three dimensions of rural digital infrastructure construction, rural industry digitalization and rural digital industry [14]. There are also scholars who construct a digital village evaluation index system based on the background of the rural revitalization strategy, from dimensions such as digital information base [15]. In addition, there are studies evaluating the level of digital village construction from county and village perspectives [16].

### 2.2 Research on high-quality agricultural development

High-quality agricultural development involves all aspects of agricultural production and operation, and is a process of overall improvement in agricultural development [17]. Studies on high-quality agricultural development have mainly focused on the level measurement and evaluation and the path of realization. For the research on level measurement and evaluation, some scholars use a single indicator to measure the level of high-quality agricultural development, such as the total factor productivity [18]. A more common practice is to construct a multidimensional evaluation index system, which can be divided into two categories according to the different dimensions. Firstly, some scholars have constructed a comprehensive evaluation framework based on the new development concept to analyze the level of high-quality agricultural development in China [19, 20]. The second is to construct an indicator system based on the connotation of high-quality development, for example, to measure the level of high-quality agricultural development of China in terms of dimensions such as green development [21]. For the research on the path of realization, most studies analyze the various factors affecting high-quality agricultural development and then propose measures to promote high-quality agricultural development. For example, scholars have identified factors such as agricultural socialization service rate [22], agricultural products quality, and agricultural production efficiency as key indicators related to high-quality agricultural development [23]. Therefore, high-quality agricultural development can be improved through measures such as upgrading the capacity to agricultural socialize services and improving agricultural production efficiency. In addition, studies have shown that in order to further promote the high-quality agricultural development of China, it is necessary to enhance the capacity for scientific and technological innovation and strengthen the use of green financial technologies [24].

### 2.3 Research on the relationship between digital village construction and high-quality agricultural development

Based on the perspective of synergy theory, digital village construction and high-quality agricultural development have a close connection. Firstly, digital village construction and high-quality agricultural development influence each other and promote each other. On the one hand, digital village construction plays an important role in promoting high-quality agricultural development. Studies have shown that village construction relying on digital technology has injected new vitality into high-quality agricultural development [25]. For example, agricultural digitalization can improve agricultural production efficiency, optimize resource allocation, and promote industrial structure upgrading [26]. In addition, digital village construction improves agricultural total factor productivity by promoting agricultural technological innovation, upgrading agricultural human capital and improving agricultural productive services [27]. On the other hand, high-quality agricultural development provides guidance and reference for digital village construction. The core of high-quality agricultural development lies in achieving sustainable agricultural development, accelerating agricultural modernization and laying the foundation for the digital transformation of agriculture [28]. Secondly, when exploring the relationship between digital village construction and high-quality agricultural development, scholars analyze the coupling and coordination mechanism of digital village construction with rural revitalization [29, 30], and carbon emission [31], respectively. The results of the studies show that digital village construction is an important strategic choice to realize the modernization of agriculture, which is closely related to agricultural production and development [32]. Another study analyze the coordination relationship between high-quality agricultural development and technological innovation, technological innovation is closely related to high-quality agricultural development, and technological innovation continuously leads high-quality agricultural development [33].

### 2.4 Research gaps and theoretical framework

In summary, the existing literature provides important references for this paper. Although existing studies have extensively explored the current situation of digital village construction and high-quality agricultural development, these studies have mainly focused on unidirectional causality and lacked research on the interactive relationship between the two. There is a bidirectional causal relationship between digital village construction and high-quality agricultural development, however, few scholars have explored the bidirectional relationship between the two, and the exploration of the coupling coordination relationship between the two is even more insufficient. Therefore, based on the existing research results, this paper intends to construct the following theoretical framework: Firstly, constructing indicator systems to scientifically evaluate the level of China’s digital village construction and high-quality agricultural development. Secondly, analyzing the coupling coordination relationship between digital village construction and high-quality agricultural development and the evolution characteristics, and exploring the regional differences. Finally, revealing the factors affecting the coupling coordination relationship and providing policy recommendations to improve the digital village construction and high-quality agricultural development.

## 3 Methodology and indicator system

### 3.1 Methodology

#### 3.1.1 Entropy weight method

The entropy weight method is a universally applied objective weighting method, which utilizes the impact of the numerical changes of each indicator on the whole to calculate the entropy value of the indicator, and then calculate the weights. In this paper, the entropy weight method is used to determine the weight of each indicator in the evaluation index system, which can effectively avoid the bias caused by subjectivity. Before the measurement, it is necessary to eliminate the influence of the index scale to standardize the indicators, and this paper refers to the method of Zhang et al. [34] to non-negativize the indicators, the specific practices are as follows:

$$Positive\;indicators:\;y_{ij}=\frac{x_{ij}-\min(x_{1j,}x_{2j}\cdots x_{nj})}{\max(x_{1j,}x_{2j}\cdots x_{nj})-\min(x_{1j,}x_{2j}\cdots x_{nj})}+0.01$$ (1)

$$Negative\;indicators:\;y_{ij}=\frac{\max(x_{1j,}x_{2j}\cdots x_{nj})-x_{ij}}{\max(x_{1j,}x_{2j}\cdots x_{nj})-\min(x_{1j,}x_{2j}\cdots x_{nj})}+0.01$$ (2)

where *x*_*ij*_ is the value of the j th indicator in the i th province, *y*_*ij*_ is the value of the j th indicator in the i th province after standardization, and max(*x*_1*j*_,*x*_2*j*_⋯*x*_*nj*_) and min(*x*_1*j*_,*x*_2*j*_⋯*x*_*nj*_) denote the maximum and minimum values of each indicator, respectively, *i* = 1,2,…*n*; *j* = 1,2,…,*m*.

Next, the entropy weight method is utilized to determine the weights of the indica-tors:

$$P_{ij}=\frac{y_{ij}}{\sum_{i=1}^{n}y_{ij}}$$ (3)

$$E_{j}=-\frac{\sum_{i=1}^{n}P_{ij}\;\ln(P_{ij})}{\ln(n)}$$ (4)

$$w_{j}=\frac{1-E_{j}}{\sum_{j=1}^{m}(1-E_{j})}$$ (5)

where *P*_*ij*_ is the weight of the sample value of the ith evaluation object under the jth indicator, *E*_*j*_ is the information entropy, and *w*_*j*_ is the weight of the jth indicator.

Finally, the composite score *S*_*i*_ of each province’s food security level is calculated:

$$S_{i}=\sum_{j=1}^{m}w_{j}\times y_{ij}$$ (6)

#### 3.1.2 Coupling coordination degree model

The coupling coordination degree model describes the interaction relationship between two or more different systems. In this paper, the coupling coordination degree model is applied to measure the coupling coordination relationship between the level of digital village construction and the level of high-quality agricultural development to analyze the interaction and coordination status of the two, and to evaluate the overall degree of development of the two [34]. The calculation formula is as follows:

$$C=\frac{2\sqrt{U_{1}U_{2}}}{U_{1}+U_{2}}$$ (7)

$$T=\alpha U_{1}+\beta U_{2}$$ (8)

$$D=\sqrt{C\times T}$$ (9)

Firstly, Formula (7) is used to calculate the coupling degree *C*, which reflects the degree of mutual influence and dependence between subsystems, *C*∈[0,1], *U*_1_、*U*_2_ respectively represents the level of digital village construction and the level of high-quality agricultural development. And then Formula (8) is used to calculate the coordination index *D*, who reflects the synergistic development between subsystems. *α*、*β* are the pending weight coefficients, this paper believes that the two are equally important, so *α* = *β* = 0.5. Finally, Formula (9) is used to calculate the degree of coupling coordination, which measures the overall level of coordination development between systems, *D*∈[0,1]. Referring to related literature [34], the coupling coordination degree of the two systems is divided into ten types, which is convenient to reflect the degree of coordination between the two systems. Table 1 shows the specific grades.

**Table 1:** Coupling coordination degree classification.

Coupling Coordination Degree	Type
[0.0,0.1)	Extreme imbalance
[0.1,0.2)	Serious imbalance
[0.2,0.3)	Moderate imbalance
[0.3,0.4)	Mid imbalance
[0.4,0.5)	Near imbalance
[0.5,0.6)	Reluctant coordination
[0.6,0.7)	Primary coordination
[0.7,0.8)	Intermediate coordination
[0.8,0.9)	Good coordination
[0.9,1.0)	High coordination

#### 3.1.3 Kernel density estimation method

Kernel density estimation is a method that uses a density function to describe the nature of data distribution. In order to demonstrate more intuitively the evolutionary pattern of the coupling coordination relationship between digital village construction and high-quality agricultural development, this paper applies this method to describe the distribution characteristics of the coupling coordination level of digital village construction and high-quality agricultural development. Then it analyzes the dynamic evolution of the coupling coordination level by comparing the kernel density curves of different years [35]. The formula is as follows:

$$f(x)=\frac{1}{nh}\sum_{i=1}^{n}K(\frac{X_{i}-\bar{x}}{h})$$ (10)

where *f*(*x*) represents the kernel density. *h* is the bandwidth, which determines the image characteristics of the kernel density curve. *n* denotes the total number of provinces. *X*_*i*_ is the level of coupling and coordination between digital village construction and high-quality agricultural development in each province. $\bar{x}$ is the mean value, and $K(\frac{X_{i}-\bar{x}}{h})$ is the kernel function.

#### 3.1.4 Spatial autocorrelation analysis method

Before applying spatial econometric modeling for analysis, the first task is to determine whether spatial correlation exists among economic units. Spatial autocorrelation mainly reveals the global spatial distribution characteristics of a certain geographical observation, which is usually characterized by the global Moran’s I index [35], whose general expression is denoted by:

$$I=\frac{n\sum_{i=1}^{n}\sum_{j=1}^{n}\omega_{ij}(x_{i}-\bar{x})(x_{j}-\bar{x})}{\sum_{i=1}^{n}\sum_{j=1}^{n}\omega_{ij}\sum_{i=1}^{n}{\left(x_{i}-\bar{x}\right)}^{2}}$$ (11)

where *I* is the global Moran’s I index, *n* is the total number of provinces, *ω*_*ij*_ is the spatial weight matrix, *x*_*i*_ and *x*_*j*_ denote the degree of coupling coordination in the ith and jth provinces, and $\bar{x}$ denotes the mean value of coupling coordination.

#### 3.1.5 Spatial econometric model

Considering that there may be some spatial correlation between the factors influencing the coupling coordination degree of digital village construction and high-quality agricultural development in different regions, and ignoring this issue may lead to biased results, this paper constructs a spatial econometric model to examine the main factors affecting the coupling coordination degree of them from a spatial perspective [36]. The general expression of the model is:

Y=ρWY+Xβ+WXθ+ε (12)

where *Y* is the dependent variable, that is, the coupling coordination degree of digital village construction and high-quality agricultural development. *X* is the explanatory variable, including the level of economic development, the degree of government intervention, technological innovation capacity, the level of human capital, financial expenditure on agriculture and upgrading of industrial structure. *W* is the spatial weight matrix, and we choose the geographic distance weight matrix, *ρ* is the spatial autoregressive coefficient, *β* is the regression coefficient, and *ε* is the random disturbance term. When *θ* = 0, the model simplifies to a spatial lag model, and when *θ*+*ρβ* = 0, the model simplifies to a spatial error model.

### 3.2 Indicator system

#### 3.2.1 Evaluation indicator system for digital village construction and high-quality agricultural development

This paper constructs the indicator system of digital village construction and high-quality agricultural development level respectively based on the scientific and systematic principles. Referring to the relevant literature and combining the connotation of digital village construction and high-quality agricultural development [14, 37–39], the indicator system of digital village construction covers digital infrastructure, digital industrial development, agricultural digitization and life digitization, and the specific indicator system is shown in Table 2. At the same time, based on the new development concept, the indicator system of the high-quality agricultural development includes agricultural innovation and development, agricultural coordination development, agricultural green development, agricultural openness development and agricultural shared development. Table 3 shows the detailed indicator system.

**Table 2:** Evaluation indicator system for digital village construction.

First-Level Indicator	Second-Level Indicator	Indicator Description	Indicator Property	Data Sources
Digital infrastructure	Rural Internet penetration rate	number of rural broadband access households/rural population	+	China Statistical Yearbook
	Rural mobile phone penetration rate	average year-end mobile phone ownership per 100 rural households	+	China Statistical Yearbook
	Rural radio and television penetration rate	number of rural radio and television subscribers/rural population	+	China Statistical Yearbook
	Rural computer penetration rate	average year-end computer ownership per 100 rural households	+	China Statistical Yearbook
Digital industry development	Digital Sales	e-commerce sales/gross regional product	+	Statistical Yearbook of Each Province
	Digital procurement	e-commerce purchases/gross regional product	+	Statistical Yearbook of Each Province
	Digital Finance	digital inclusive finance development index	+	Institute of Digital Finance Peking University
	Number of rural express outlets	data directly available	+	China Rural Statistical Yearbook
Digitization of agriculture	Gross power of agricultural machinery per capita	total power of agricultural machinery/number of people working in the primary sector	+	China Statistical Yearbook
	Per capita value of agriculture, forestry and fisheries	gross output value of agriculture, forestry, animal husbandry and fishery/number of persons employed in the primary sector	+	China Statistical Yearbook
	Rural electricity consumption per capita	total rural electricity consumption/rural population	+	China Rural Statistical Yearbook
	Number of agrometeorological observation stations	data directly available	+	China Rural Statistical Yearbook
Digitization of life	Engel’s coefficient for rural households	data directly available	-	China Rural Statistical Yearbook
	Level of consumption of digitized services	per capita transportation and communication consumption expenditure of rural residents	+	China Rural Statistical Yearbook
	Income of the rural population	per capita disposable income of rural residents	+	China Rural Statistical Yearbook
	Consumption of the rural population	per capita consumption expenditure of rural residents	+	China Rural Statistical Yearbook

**Table 3:** Evaluation indicator system for high-quality agricultural development.

First-Level Indicator	Second-Level Indicator	Third-Level Indicator	Indicator Description	Indicator Properties	Data Sources
Innovative development of agriculture	Innovative foundations	Investment in agricultural science and technology activities	Internal expenditures on R&D* (gross output value of agriculture, forestry, livestock and fisheries/gross regional product)	+	China Statistical Yearbook
		Degree of agricultural mechanization	Total power of agricultural machinery/cultivated area	+	China Statistical Yearbook
	Efficiency gains	Labor productivity	Output of primary industry/number of employees in primary industry	+	China Rural Statistical Yearbook
		Food output rate	Total grain production/sown area of grain	+	China Rural Statistical Yearbook
		Effective irrigation rate	Effective irrigated area/total sown area of crops	+	China Rural Statistical Yearbook
Coordination development of agriculture	Industrial coordination	Industrial structure optimization index	1 - (gross value of agricultural output/gross value of agricultural, forestry, livestock and fisheries output)	+	China Statistical Yearbook
		Cultivation diversification index	(Total sown area of crops—sown area of grain)/Total sown area of crops	+	China Statistical Yearbook
	Urban and rural coordination	Comparison of income of urban and rural residents	Per capita disposable income of urban residents/per capita disposable income of rural residents	-	China Statistical Yearbook
		Comparison of consumption between urban and rural residents	Per capita consumption expenditure of urban residents/per capita consumption expenditure of rural residents	-	China Statistical Yearbook
		Strength of the urban-rural dichotomy	Comparative labor productivity in secondary and tertiary industries/comparative labor productivity in primary industries	-	China Statistical Yearbook
Green development of agriculture	Low carbon production	Pesticide application intensity	Pesticide application/total sown area of crops	-	China Statistical Yearbook
		Fertilizer application intensity	Fertilizer application/total sown area of crops	-	China Statistical Yearbook
	Environmental protection	Intensity of plastic film use in rural areas	Rural plastic film use/total sown area of crops	-	China Statistical Yearbook
		Forest cover rate	Data directly available	+	China Statistical Yearbook
		Crop damage rate	Area damaged/area affected	-	China Statistical Yearbook
Open development of agriculture	Openness	Dependence on foreign trade in agricultural products	Total agricultural exports and imports/gross regional product	+	Statistical Yearbook of Each Province
Shared development of agriculture	Welfare sharing	Percentage of social security in rural areas	Number of rural residents with minimum subsistence allowance/rural population	-	Statistical Yearbook of Each Province
		Level of rural health care	Rural health technicians per 1,000 population	+	Statistical Yearbook of Each Province
	Achievement sharing	Engel’s coefficient for rural households	Data directly available	-	China Rural Statistical Yearbook
		Per capita disposable income of rural residents	Data directly available	+	China Rural Statistical Yearbook

#### 3.2.2 Factors affecting the degree of coupling and coordination between digital village construction and high-quality agricultural development

Referring to relevant literature [40–42], the level of coupling and coordination between digital village construction and high-quality agricultural development is selected as the dependent variable. And the level of economic development, the degree of government intervention and so on are the explanatory variables for econometric analysis. Table 4 demonstrates the variable descriptions and descriptive statistics of each factor.

**Table 4:** Description of variables influencing the degree of coupling coordination.

Variable Classification	Variable Name	Variable Symbol	Variables Description	Data Sources	Obs	Mean	Standard Deviation
Dependent variable	Level of coupling coordination	D	Degree of coupling coordination	Calculated from Eq (9)	372	0.5760	0.1033
Explanatory variable	Level of economic development	eco	GDP per capita (logarithmic)	China Statistical Yearbook	372	10.8563	0.4620
	Level of government intervention	gov	Proportion of fiscal expenditure to GDP	China Statistical Yearbook	372	0.2906	0.2055
	Technological innovation capacity	tec	Number of patent applications granted (logarithmic)	Statistical Yearbook of Each Province	372	10.1567	1.6299
	Level of human capital	hum	Proportion of persons with tertiary education and above	Statistical Yearbook of Each Province	372	0.1501	0.0775
	Financial expenditure on agriculture	agr	Proportion of agricultural fiscal expenditure	China Rural Statistical Yearbook	372	0.1151	0.0341
	Upgrading of industrial structure	ind	Proportion of added value of tertiary industry to GDP	China Statistical Yearbook	372	0.4989	0.0887

### 3.3 Data sources

This study focuses on 31 provinces (municipalities and autonomous regions) in China (excluding Hong Kong, Macao and Taiwan) and examines the time span of 2011–2022. Hong Kong, Macao and Taiwan are not included in the study given the large amount of missing data for these regions, so we only selected data from 31 provinces (municipalities and autonomous regions) in mainland China. The data for this study are mainly derived from the China Statistical Yearbook, the China Rural Statistical Yearbook, the statistical yearbooks of each province and Institute of Digital Finance Peking University. The detailed sources of each indicator are reflected in the table. By searching the statistical yearbook, the data of the indicators that can be obtained directly are organized, and the data that need to be calculated are organized and calculated through the relevant formulas. Some missing data are supplemented and refined by the interpolation method.

## 4 Results and analysis

### 4.1 Analysis of the level of digital village construction and high-quality agricultural development

#### 4.1.1 Temporal evolution analysis

According to the indicator system and measurement formula in the previous section, we measure the scores of the level of digital village construction and high-quality agricultural development from 2011 to 2022. In order to facilitate further analysis, the study area is divided into the eastern region, central region and western region according to the standard of China’s economic belt division. Table 5 shows the mean values of the scores.

**Table 5:** Score on the level of digital rural development and high-quality agricultural development.

Year	Level of Digital Village Construction				Level of High-quality Agricultural Development			
	Eastern regions	Central regions	Western regions	National regions	Eastern regions	Central regions	Western regions	National regions
2011	0.4327	0.2268	0.157	0.2722	0.5422	0.395	0.3093	0.4155
2012	0.4301	0.2307	0.1673	0.276	0.5359	0.3971	0.3162	0.4164
2013	0.3989	0.2246	0.1636	0.2624	0.4923	0.3438	0.2784	0.3715
2014	0.3905	0.219	0.1666	0.2587	0.5335	0.4077	0.3168	0.4193
2015	0.3941	0.2262	0.1783	0.2662	0.537	0.3983	0.3169	0.4174
2016	0.3931	0.2362	0.1899	0.2731	0.5402	0.4005	0.3181	0.4196
2017	0.3925	0.2336	0.1824	0.2695	0.5364	0.3976	0.3227	0.4189
2018	0.3978	0.2398	0.1892	0.2756	0.538	0.4029	0.3178	0.4196
2019	0.3874	0.2386	0.1938	0.2733	0.5519	0.424	0.3439	0.4399
2020	0.4629	0.2953	0.2498	0.336	0.5309	0.4313	0.3454	0.4359
2021	0.4512	0.2853	0.2458	0.3274	0.5439	0.4285	0.3479	0.4401
2022	0.4453	0.281	0.2379	0.3214	0.5392	0.4321	0.3473	0.4395

From 2011 to 2022, the level of digital village construction and high-quality agricultural development have both increased. The level of digital village construction grows from 0.2722 to 0.3214, an increase of 18.07%. And the level of high-quality agricultural development grows from 0.4155 to 0.4395, an increase of 5.78%. These results are basically consistent with the research of scholars such as Lu et al. [43] and Xiang et al. [20]. Although the level of digital village construction and high-quality agricultural development are at a low stage, both are in an upward trend. Digital village construction has seen a large increase and rapid development. As one of the important strategic directions for rural revitalization, the digital village construction strategy has been developing in recent years, and with the continuous improvement of digital infrastructure in rural areas, the digital economy and digital governance in rural areas have achieved significant results. At present, China’s agriculture is following the new development concept and gradually transitioning to the stage of high-quality development. At the same time, the digital transformation of agriculture has accelerated, and the industrial structure has been optimized. So the level of high-quality agricultural development has steadily increased.

#### 4.1.2 Spatial distribution patterns

From a spatial point of view, China’s digital village construction and high-quality agricultural development level show spatial imbalance. The digital village construction and high-quality agricultural development in the eastern region far exceed the national average, while the central and western regions are relatively backward, which shows a decreasing trend from east to west. Mainly due to the high level of economic development in the eastern region. And with the establishment of some digital village pilot areas in the eastern region, the digital infrastructure is gradually improved, which lays a good foundation for the construction of digital villages. Li and Wen’s research also shows that the eastern region has a higher level of digital village construction due to a higher level of information infrastructure development, while the western region has a slower start in digital village construction due to the lack of maturity of the basic conditions [44]. In recent years, thanks to the promotion of strategies such as "East data, West computing", the central and western regions have gradually been introduced into the process of digital development, and a series of policies have been implemented to promote digital transformation and continuously improve the level of digital village construction. In addition, the eastern region has an advantageous geographical location, rich agricultural resources, a high level of agricultural mechanization, and large-scale agricultural development, all of which have contributed to the high-quality agricultural development. The central and western regions are constrained by factors such as the resource environment and economic development, and the high-quality agricultural development is relatively backward. But with the continuous improvement of the level of digital village construction, the agricultural infrastructure is constantly being strengthened, and agricultural production methods are transforming to digitalization, which provides a good environment for the high-quality agricultural development.

### 4.2 Analysis of the level of coupling coordination relationship of digital village construction and high-quality agricultural development

Under the premise of measuring the level of China’s digital village construction and high-quality agricultural development, the degree of coupling coordination between them is measured by the coupling coordination degree model. Table 6 shows the results. Observed in time, from 2011 to 2022, the coupling coordination degree of them realizes an increase from 0.5645 to 0.6040, and the coupling coordination level is also raised from reluctant coordination to primary coordination. As far as the spatial distribution is concerned, the coupling coordination level of them in different regions shows variability. For example, the coupling coordination degree of the eastern region exceeds the national average, and the coupling coordination type stays at the primary coordination level. According to the previous analysis, the level of digital village construction and high-quality agricultural development in the eastern region are in the leading position. So, based on good digital infrastructure construction and rich agricultural resources, the level of digital village construction and high-quality agricultural development in the eastern region are jointly improved and coordinated. While the central and western regions are still in the stage of reluctant coordination. In recent years, the gap between the regions has been gradually narrowing based on national policy inclination and the development of digital logistics in the west. Although the level of coupling and coordination between digital village construction and high-quality agricultural development is gradually improving nationwide, the overall degree of coupling and coordination needs to be further improved.

**Table 6:** Degree and type of coupling coordination between digital village construction and high-quality agricultural development.

Year	Degree of Coupling Coordination				Type of Coupling Coordination			
	Eastern regions	Central regions	Western regions	National regions	Eastern regions	Central regions	Western regions	National regions
2011	0.6855	0.5448	0.4632	0.5645	Primary coordination	Reluctant coordination	Near imbalance	Reluctant coordination
2012	0.6836	0.5485	0.4743	0.5688	Primary coordination	Reluctant coordination	Near imbalance	Reluctant coordination
2013	0.6571	0.5261	0.4563	0.5465	Primary coordination	Reluctant coordination	Near imbalance	Reluctant coordination
2014	0.6668	0.5453	0.4728	0.5616	Primary coordination	Reluctant coordination	Near imbalance	Reluctant coordination
2015	0.6713	0.5466	0.4814	0.5664	Primary coordination	Reluctant coordination	Near imbalance	Reluctant coordination
2016	0.6719	0.5528	0.49	0.5716	Primary coordination	Reluctant coordination	Near imbalance	Reluctant coordination
2017	0.6706	0.55	0.4857	0.5687	Primary coordination	Reluctant coordination	Near imbalance	Reluctant coordination
2018	0.6734	0.5552	0.4905	0.573	Primary coordination	Reluctant coordination	Near imbalance	Reluctant coordination
2019	0.6728	0.5614	0.5036	0.5793	Primary coordination	Reluctant coordination	Reluctant coordination	Reluctant coordination
2020	0.6994	0.5952	0.5366	0.6104	Primary coordination	Reluctant coordination	Reluctant coordination	Primary coordination
2021	0.6994	0.5887	0.5353	0.6078	Primary coordination	Reluctant coordination	Reluctant coordination	Primary coordination
2022	0.6946	0.587	0.5304	0.604	Primary coordination	Reluctant coordination	Reluctant coordination	Primary coordination

**Fig 1:**
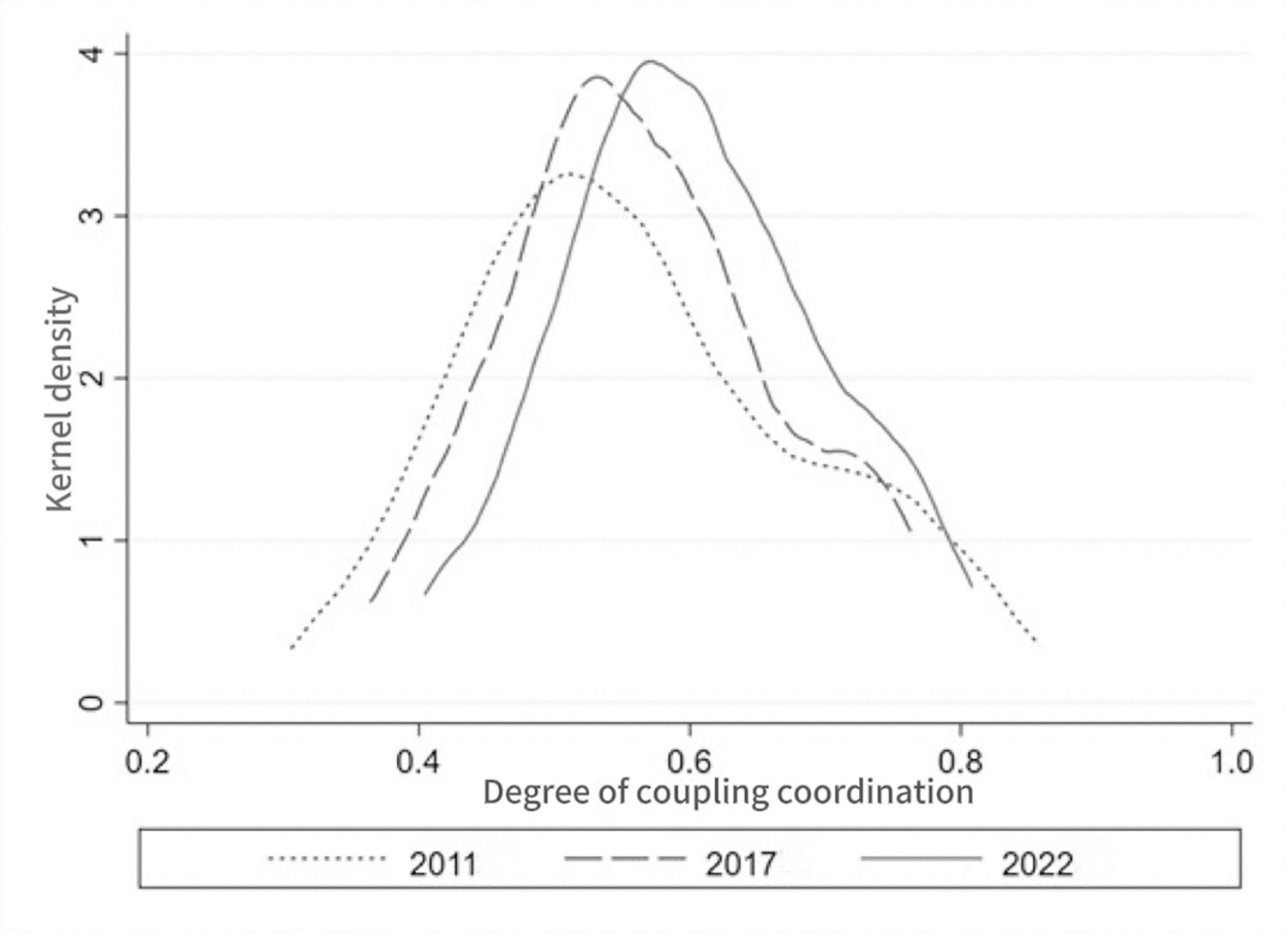
Kernel density distribution of coupling coordination degree.

In order to dynamically demonstrate the evolutionary characteristics of the coupling coordination relationship between digital village construction and high-quality agricultural development in China, we map the kernel density distribution of the coupling coordination degree using Stata15 software for the years of 2011, 2017 and 2022. And Fig 1 illustrates the kernel density plot. From the position of the kernel density distribution map, the kernel density curve of the coupling and coordination degree in 2011–2022 shifted to the right as a whole, which indicates an increase in the number of high-value regions and a decrease in the number of low-value regions. This indicates that the coupling and coordination level of digital village construction and high-quality agricultural development in China shows a gradual increase during the study period. Specifically, from 2011 to 2022, the height of the wave peak of the kernel density curve gradually becomes higher, and the width of the wave peak becomes smaller, which indicates that the coupling and coordination level of them tends to be centralized, and the differences between regions are gradually decreasing, showing a dynamic convergence trend. This indicates that during the study period, the differences in the coupling coordination level of digital village construction and high-quality agricultural development among regions in China have gradually narrowed, and the coupling coordination level has realized common improvement. Wang and Tang have studied the coupling coordination degree of digital village and green and high-quality agricultural development in China, and the results show that the overall coupling coordination degree is around 0.55 in 2010–2019, with variability among different regions and the difference between the eastern region and the central and western regions gradually increases [45]. However, different from their findings, this paper demonstrates the dynamic evolution characteristics of the coupling coordination degree of the two through the kernel density plot, and the results show that the inter-regional differences show a gradual decrease. Possible reasons include that this study explores spatial differences from a perspective of dynamic evolution. Additionally, differences in the study period and indicator measurements are also important factors.

### 4.3 Analysis of factors influencing the coupling coordination degree between digital village construction and high-quality agricultural development

#### 4.3.1 Spatial correlation analysis

Firstly, we use Stata15 software to calculate the global Moran’s I index of the coupling coordination degree of digital village construction and high-quality agricultural development from 2011 to 2022 to test the spatial correlation. Table 7 shows the specific results. As can be seen in Table 7, all the Moran’s I indices of the coupling coordination level have passed the 1% significance level test, which indicates that the coupling coordination degree of China’s digital village construction and high-quality agricultural development presents a significant positive correlation in space, so this paper can be analyzed by using the spatial econometric model.

**Table 7:** Results of global Moran’s I index.

Year	Moran’s I	P-value	Year	Moran’s I	P-value
2011	0.164	0.000	2017	0.165	0.000
2012	0.164	0.000	2018	0.17	0.000
2013	0.175	0.000	2019	0.157	0.000
2014	0.17	0.000	2020	0.154	0.000
2015	0.171	0.000	2021	0.156	0.000
2016	0.17	0.000	2022	0.156	0.000

#### 4.3.2 Spatial econometric model selection

In order to select the appropriate spatial econometric model, this study further utilizes various methods such as LM test, Wald test, LR test, etc., and the specific results are shown in Table 8. Analyzing the data in Table 8, it can be seen that except for the LM-Lag failing to pass the test of significance, the others pass the test of significance at the 1% level, and the statistic result of Hausman’s test is -85.42, showing a more complex situation. After simulation and analysis, it is found that this is mainly due to the fact that the basic assumption of the random effects model, i.e., the asymptotic assumption, cannot be satisfied. Referring to the related literature, it can be seen that the fixed effect model should be used in this study [46]. Meanwhile, by comparing individual fixed effects, time fixed effects and two-way fixed effects, it is found that two-way fixed effects have the largest Log-likelihood value and the best effect. Therefore, based on the above test results, this paper finally adopts the two-way fixed Spatial Durbin Model (SDM) to analyze the influencing factors of the coupling and coordination of digital village construction and high-quality agricultural development.

**Table 8:** Results of the spatial measurement model selection test.

Test Methods	Statistic	P-value
LM-Err	133.234	0.000
Robust LM-Err	138.853	0.000
LM-Lag	1.071	0.301
Robust LM-Lag	6.690	0.010
Wald-Err	44.100	0.000
Wald-Lag	47.720	0.000
LR-Err	43.300	0.000
LR-Lag	45.420	0.000

#### 4.3.3 Analysis of influencing factors

In order to further assess the robustness of the model, this paper also constructs the Spatial Error Model (SEM), Spatial Lag Model (SLM) and Spatial Durbin Model (SDM) of the influencing factors of the coupling degree of coordination between the two, and the specific results are shown in Table 9. The following conclusions can be drawn: First of all, the SDM model has the best effect in spatial econometric analysis of the coupling degree of coordination between the digital village construction and the high-quality agricultural development. By comparing the test results of different models, it can be found that the SDM model has the largest Log-likelihood value, which indicates that it is reasonable and appropriate to use the SDM model to analyze the influencing factors of the degree of coupling and coordination between the two, and this conclusion is consistent with the previous test results. Secondly, the estimation results of the two-way fixed-effects SDM model show that the coefficient estimates of the spatially lagged terms of the economic development level (W*eco), technological innovation capacity (W*tec) and agricultural financial expenditure (W*agr) pass the significance test. This indicates that the level of economic development, technological innovation capacity and agricultural financial expenditure in neighboring regions have a significant positive spatial effect on the local coupling coordination, while the spatial effect of the level of government intervention, human capital level and industrial structure upgrading in neighboring regions on the local coupling coordination is not significant. Finally, there is a significant spatial spillover effect of the coupling coordination degree of digital village construction and high-quality agricultural development. The spatial autoregressive coefficient in the SDM model is -0.5338 and is significant at the 5% level, indicating that for every 1% increase in the coupling coordination degree of neighboring regions, the coupling coordination degree of the local region will decrease by 53.38%. This is mainly due to the fact that the coupling coordination degree of the two in each province is still at a low stage, and the radiation and demonstration effects of the region with high coupling coordination on the neighboring low-value areas have not yet been fully realized. In a society with limited economic resources, the rapid improvement of the digital village construction and the high-quality agricultural development will be attractive to the human and material resources of neighboring regions, creating a siphon effect and showing a negative spatial spillover effect.

**Table 9:** Results of the spatial econometrics model of coupling coordination.

Variable	SEM	SLM	SDM
eco	0.1669*** (9.13)	0.1545*** (7.95)	0.1145*** (5.04)
gov	-0.0012 (-0.03)	0.0051 (0.13)	-0.0076 (-0.21)
tec	0.0111*** (2.68)	0.0096** (2.31)	0.0047 (1.17)
hum	-0.2247*** (-3.97)	-0.2228*** (-3.91)	-0.1673*** (-3.06)
agr	0.3117*** (3.98)	0.2842*** (3.65)	0.2822*** (3.58)
ind	0.1882*** (3.82)	0.1761*** (3.55)	0.1539*** (3.04)
W*eco	-	-	0.4084*** (3.81)
W*gov	-	-	-0.4144 (-1.40)
W*tec	-	-	0.0591* (1.88)
W*hum	-	-	-0.0743 (-0.22)
W*agr	-	-	1.2261** (2.44)
W*ind	-	-	0.4667 (1.43)
Spatial error regression coefficient λ	-0.3465 (-1.54)	-	-
Spatial autoregression coefficient ρ	-	0.1028 (0.64)	-0.5338** (-2.51)
R^2^	0.4037	0.3977	0.3929
LogL	1013.9004	1012.8375	1035.5482

Note

***, **, * denote significant at 1%, 5%, 10% level, respectively.

In order to more accurately analyze the influence of each factor on the level of coupling coordination, the spatial effects of each influencing factor are further decomposed with the help of partial differential method, and the specific results are shown in Table 10. In terms of the total effect, the influence of the level of economic development, technological innovation capacity, agricultural financial expenditure and industrial structure upgrading on the coupling coordination level of digital village construction and high-quality agricultural development is significantly positive, while the spatial total effect of the degree of government intervention and the level of human capital on the coupling coordination level is not significant. This indicates that the level of economic development, technological innovation capacity, agricultural financial expenditure and industrial structure upgrading have a significant role in promoting the coupling of digital village construction and high-quality agricultural development.

**Table 10:** Decomposition of spatial effects of coupling coordination.

Variable	Direct Effect	Indirect Effect	Total Effect
eco	0.1079*** (4.43)	0.2309*** (2.94)	0.3388*** (4.92)
gov	0.0004 (0.01)	-0.2887 (-1.42)	-0.2883 (-1.39)
tec	0.0038 (0.95)	0.0395* (1.89)	0.0432** (2.16)
hum	-0.1682*** (-3.12)	0.0211 (0.09)	-0.1471 (-0.66)
agr	0.2593*** (3.28)	0.7084** (2.01)	0.9677*** (2.83)
ind	0.1504*** (2.89)	0.2841 (1.19)	0.4345** (1.79)

Note

***, **, * denote significant at 1%, 5%, 10% level, respectively.

The level of economic development is a key factor to promote the coupling coordination development of digital village construction and high-quality agricultural development, showing a significant facilitating effect and positive spillover effect. In terms of the direct impact of the level of economic development, its effect coefficient on the degree of coupling and coordination of them in local region is 0.1079, and it is established at the 1% significance level. This fully indicates that the increase in the level of economic development has a significant role in promoting the coupling coordination development of digital village construction and high-quality agricultural development in local region. Under the condition of assuming that other variables remain unchanged, every 1% increase in the economic level will directly promote the coupling coordination development of local region by 10.79%. In terms of the indirect effect of the level of economic development, the coefficient of its influence on the degree of coupling and coordination of them in neighboring regions is 0.2309, and it is established at a significant level of 1%. This indicates that the increase in the level of local economic development has a significant driving effect on the increase in the degree of coupling and coordination of them in neighboring regions. Under the premise of keeping other variables constant, for every 1% increase in the economic level, the coupling coordination degree of digital village construction and high-quality agricultural development in neighboring regions increases by 23.09%. On the one hand, the improvement of the economic development level attracts more talents and material resources, which lays a material foundation for the digital village construction. On the other hand, the improvement of the economic development level is accompanied by the continuous improvement of the agricultural infrastructure, and the level of the agricultural mechanization shows a significant increase, which accelerates the digital transformation of agriculture, and promotes the coupling and coordination of the digital village construction and the high-quality agricultural development.

The direct and indirect effects of the degree of government intervention on the coupling and coordination of digital village construction and high-quality agricultural development do not pass the significance test, indicating that government intervention does not have a significant impact on the coupling and coordination of digital village construction and high-quality agricultural development in local region and neighboring regions.

The coefficient of the spatial spillover effect of technological innovation capacity on the coupling coordination level of digital village construction and high-quality agricultural development is 0.0395, which passes the test at the 10% significance level, indicating that the improvement of the local technological innovation capacity positively promotes the improvement of the coupling coordination degree in the neighboring regions. Under the condition of assuming that other variables remain unchanged, every 1% increase in technological innovation capacity will promote 3.95% increase in the coupling and coordination degree in neighboring regions. The direct impact effect of technological innovation capacity does not pass the significance test, indicating that the impact of technological innovation capacity on the coupling coordination development of local digital village construction and high-quality agricultural development is not significant. Theoretically, technological innovation is the core driving force of digital village construction and high-quality agricultural development. However, in practice, technological innovation activities often lead to an imbalance in the allocation of funds due to the high requirements of capital investment, which limits the wide application of technological innovation in digital village construction and high-quality agricultural development. At present, China’s digital village construction is in the exploratory stage, and there is still a vast space for improvement of new generation information technology in promoting the synergistic promotion of digital village construction and high-quality agricultural development.

The direct effect of human capital level on the degree of coupling and coordination of digital village construction and high-quality agricultural development is -0.1682, and it is significant at 1% level, which indicates that human capital level has a significant inhibitory effect on the degree of coupling and coordination of local region. Under the condition of assuming that other variables remain unchanged, for every 1% increase in the level of human capital, the degree of coupling coordination will decrease by 16.82%. It indicates that the increase in the level of human capital is not conducive to the development of coupling and coordination in local region, which may be due to the fact that there are some structural deficiencies in the supply of human resources, and the human capital is not yet fully adapted to the needs of digital village construction and high-quality agricultural development. In addition, the underutilization of highly qualified personnel is also an important reason. The indirect effect of human capital level does not pass the significance test, indicating that the spatial spillover effect of human capital level on the coupling and coordination degree of digital village construction and high-quality agricultural development in neighboring regions is not obvious, probably because the study time interval selected is relatively short, and the level of human capital has not changed too much in this interval, which has a certain lag, and the spillover effect on the coupling and coordination degree of digital village construction and high-quality agricultural development has not been played out.

Agricultural financial expenditure, as one of the key factors, has a significant impact on the coupling coordination relationship between digital village construction and high-quality agricultural development, showing a significant facilitating effect and positive spillover effect. The effect of agricultural financial expenditure on the coupling and coordination degree in local region is 0.2593 and significant at 1% level. And the indirect effect on the coupling and coordination degree in neighboring regions is 0.7084 and significant at 5% level. Under the condition of assuming that other variables remain unchanged, every 1% increase in agricultural financial expenditure will directly promote the coupling and coordination degree of local region by 25.93%, and indirectly promote the coupling and coordination degree in neighboring regions by 70.84%. At present, China is in the transition stage from traditional agriculture to modern agriculture, and increasing financial expenditure on agriculture is conducive to solving the difficulties and challenges encountered in the transition. On the one hand, increasing agricultural financial expenditure is conducive to improving the level of rural economic development, accelerating the process of agricultural digital transformation, and providing stronger support for the construction of digital villages. On the other hand, agricultural financial expenditure is committed to improving the financial support for agricultural infrastructure and skills training of workers, which in turn improves the production conditions of agriculture, improves the efficiency of agricultural production, and promotes the high-quality agricultural development.

The direct effect of industrial structure upgrading on the coupling coordination degree of local digital village construction and high-quality agricultural development is 0.1504, and it passes the significance test at 1% level. This indicates that industrial structure upgrading has a significant promotion effect on the coupling coordination degree of digital village construction and high-quality agricultural development. Under the premise of assuming that other variables remain unchanged, for every 1% increase in industrial structure upgrading, the degree of coupling and coordination will increase by 15.04%. Upgrading of industrial structure means that the development level and efficiency of the tertiary industry have been improved. In recent years, the national policy tends to develop the information technology service industry and other directions, the digital industry has been rapidly cultivated. And the process of digital transformation of agriculture has been accelerated, which has transformed the traditional agricultural production structure, and provided industrial and economic support for the digital village construction and high-quality agricultural development. The spatial spillover effect of industrial structure upgrading is positive, but does not pass the significance test, which indicates that industrial structure upgrading does not have a significant impact on the coupling coordination development of the two in neighboring regions. Wang and Tang’s research indicates that factors such as the level of economic, agricultural financial input, industrial structure, and innovation level play a promotive role in the coupling coordination of digital village and green and high-quality agricultural development in China [45], which is basically consistent with the conclusions of this study. The difference lies in that this study explores the spatial spillover effects of various influencing factors from a spatial perspective, whereas Wang and Tang’s research examines the changes in the influence of factors from a temporal dimension.

#### 4.3.4 Robustness test

In order to test the robustness of the model results, the geographic distance weight matrix was replaced with the economic distance weight matrix and the results obtained are shown in Table 11. From the results, it can be seen that the direction of the influence of each factor remains constant and the degree of significance does not change much, which proves the robustness of the estimation results.

**Table 11:** Robustness test results.

Variables	Weight Matrix of Economic Distance
eco	0.1486***(8.06)
gov	0.0140(0.36)
tec	0.0010(2.36)
hum	-0.1917***(-3.36)
agr	0.2358***(2.86)
ind	0.2056***(4.14)
Spatial autoregression coefficient ρ	-0.652*(-0.67)
R^2^	0.4064
LogL	1021.5306

Note

***, **, * denote significant at 1%, 5%, 10% level, respectively.

## 5 Discussion

In this study, we analyze the coupling coordination relationship between digital village construction and high-quality agricultural development in China. Specifically, we first establish indicator systems to measure the level of digital village construction and the level of high-quality agricultural development, then explain the interaction between the two using the coupling coordination degree model, and finally identify the key influencing factors using the spatial econometrics model and propose policy recommendations.

Traditional agriculture faces problems such as low efficiency and inadequate resource utilization, and more and more scholars have noticed the importance of digitization for agricultural development. Accelerating the integration of digital technology and agriculture is the key to promoting the high-quality development of agriculture [28]. Keefe et al. show that digitization can improve Indonesia’s agricultural industry chain resilience [47], and Song et al. argue that the digital economy significantly promotes high-quality agricultural development through labor transfer [48]. In addition, Njuguna et al. argue that the development of smallholder agriculture in the African region faces many challenges, and explore the ways in which digital tools change the impact of agriculture, using Kenya as an example [49]. Existing studies provide good insights for us to conduct this study.

Based on previous research, the main findings of this study are as follows: Firstly, the level of digital village construction and high-quality agricultural development in China have increased from 2011 to 2022, which is consistent with the findings of Cao [14] and Wang [19]. In addition, this study finds that the increase in the level of digital village construction is greater. Secondly, from 2011 to 2022, the degree of coupling and coordination between China’s digital village construction and high-quality agricultural development have increased overall. There are differences between regions, and the coupling coordination level in the eastern region is better than that in the central and western regions, which is mainly related to the high economic level and rich agricultural resources in the eastern region. Regional differences are gradually narrowing, and all regions are realizing a common improvement in the coupling coordination level. This conclusion differs from the findings of Wang and Tang, they have studied the coupling coordination degree of digital village and green and high-quality agricultural development in China, and the results show that the overall coupling coordination degree is around 0.55 in 2010–2019, with variability among different regions and the difference between the eastern region and the central and western regions gradually increases [45]. Possible reasons include that this study explores spatial differences from a perspective of dynamic evolution. Additionally, differences in the study period and indicator measurements are also important factors. Finally, there is a significant spatial correlation between the coupling coordination level of digital rural construction and high-quality agricultural development in China, and factors such as the level of economic development have an impact on the coupling coordination relationship between the two. This is also consistent with Wang and Tang’s study. The difference lies in that this study explores the spatial spillover effects of various influencing factors from a spatial perspective, whereas Wang and Tang’s research examines the changes in the influence of factors from a temporal dimension. In addition, this paper finds that the level of human capital has a negative inhibitory effect on the coupling coordination relationship between the two, and the effect of government intervention is insignificant.

Scientific understanding of the coupling coordination relationship between digital village construction and high-quality agricultural development is of great significance in promoting the common improvement of them. There are numerous studies on digital village construction and high-quality agricultural development, but through literature comparison and analysis, we find that the perspective of the existing studies is single, and the coupling coordination relationship between the two is insufficiently explored. Therefore, this study attempts to make a preliminary exploration that. This study comprehensively analyzes the internal mechanism and evolutionary characteristics of the coordination relationship between the two, and reveals the key factors affecting the coordination relationship. On the one hand, this paper provides literature references for the study of the interaction between the two, enriches and supplements the research in the field of digital village and high-quality agricultural development, and has important theoretical significance. On the other hand, by analyzing the factors affecting the coordination relationship between the two, this paper provides a scientific basis for the formulation of relevant policies and a practical reference for promoting the construction of digital village and high-quality agricultural development in China.

However, there are some limitations in this paper. Firstly, the indicator system of digital village construction and high-quality agricultural development may not be comprehensive enough. In order to solve this limitation, in the future, more indicators can be selected from multiple perspectives on this basis, and a more comprehensive indicator system can be established so as to draw more comprehensive conclusions. Secondly, the research object of this study is from the provinces of China, and the data are mainly from the China Statistical Database. Future research can extend the research scope to the city level of China as well as other countries. Finally, the factors affecting the coupling coordination degree may be incomplete and selected with subjectivity. Therefore, future research can use more scientific methods to analyze more influencing factors and get more comprehensive conclusions.

## 6 Conclusions and policy recommendations

### 6.1 Conclusion

In this paper, on the basis of measuring the level of digital village construction and high-quality agricultural development in all provinces of China from 2011 to 2022, we use the coupling coordination model to measure the coupling coordination level of the two, and construct a spatial Durbin model to analyze the influencing factors of the coupling coordination relationship between them. The main conclusions are as follows: Firstly, during the study period, the level of digital village construction and the level of high-quality agricultural development in all provinces of China have improved as a whole. The digital village construction and high-quality agricultural development in the eastern region far exceed the national average, while the central and western regions are relatively backward, which shows a decreasing trend from east to west. Secondly, during the study period, the degree of coupling coordination between digital village construction and high-quality agricultural development in China has increased as a whole. There is variability in the level of coupling coordination between regions, which is manifested in the fact that the eastern region is higher than the central and western regions. But from a dynamic evolutionary perspective, the regional variability is gradually shrinking. Thirdly, the level of economic development and agricultural financial expenditures are important factors affecting the degree of coupling coordination of them, which not only have a positive facilitating effect, but also a positive spatial spillover effect. The effects of industrial structure upgrading and technological innovation capacity are also significant, with positive facilitating effects and positive spatial spillover effects, respectively. The level of human capital has a negative inhibitory effect, and the effect of government intervention is not significant.

### 6.2 Policy recommendations

Based on the previous research and analysis, in order to promote the coupling and coordination of China’s digital village construction and high-quality agricultural development, we put forward the following policy recommendations: Firstly, it is necessary to strengthen inter-regional cooperation and exchanges. At present, there is still a certain gap between the digital village construction and the level of high-quality agricultural development among various regions in China. Thus, proactive inter-regional collaboration is essential to foster mutual growth. The governments should formulate differentiated development policies based on the unique requirements of the eastern, central and western regions. For example, it should set up agricultural technology innovation platforms and other scientific research institutions to promote the dissemination and application of agricultural technology innovations within these regions. Furthermore, it is also desirable to share advanced technologies and successful experiences from the eastern region to the central and western regions through the organization of exchanges and studies, demonstrations and promotions. Secondly, the government is expected to reshape its public service functions and take the lead. The government should provide sufficient financial support to improve the level of digital development in rural areas. For example, digital agricultural production bases can be established to improve agricultural production efficiency and optimize farmland management by using technologies such as big data and artificial intelligence. Then it is necessary to establish a digital marketing platform to improve the sales efficiency and value of agricultural products through rural e-commerce. In addition, attention should also be directed towards setting up digital agriculture demonstration bases to encourage more farmers to adopt digital agricultural production models, thereby propelling high-quality agricultural development. Thirdly, it is necessary to cultivate high-quality talents and strengthen technological innovation capabilities. For example, organizing digital skills training for farmers and agricultural enterprises to improve their digital literacy. Compared with urban areas, it is difficult for rural areas to keep up with the development of the digital age, and the digital divide between urban and rural areas may lead to slow digital development in rural areas. Therefore, tailored training programs focusing on rural production and daily life contexts should be implemented to facilitate easier access to digital technologies and enhance digital skills among farmers. Additionally, financial support and policy incentives can be established to encourage farmers and enterprises to innovate in the field of digital agriculture.

## Supporting information

S1 Data (ZIP)
